# Drift–diffusion models for multiple-alternative forced-choice decision making

**DOI:** 10.1186/s13408-019-0073-4

**Published:** 2019-07-03

**Authors:** Alex Roxin

**Affiliations:** 1 Centre de Recerca Matemàtica, Bellaterra, Spain; 2 Barcelona Graduate School of Mathematics, Barcelona, Spain

**Keywords:** Decision making, Networks, Winner-take-all

## Abstract

The canonical computational model for the cognitive process underlying two-alternative forced-choice decision making is the so-called drift–diffusion model (DDM). In this model, a decision variable keeps track of the integrated difference in sensory evidence for two competing alternatives. Here I extend the notion of a drift–diffusion process to multiple alternatives. The competition between *n* alternatives takes place in a linear subspace of $n-1$ dimensions; that is, there are $n-1$ decision variables, which are coupled through correlated noise sources. I derive the multiple-alternative DDM starting from a system of coupled, linear firing rate equations. I also show that a Bayesian sequential probability ratio test for multiple alternatives is, in fact, equivalent to these same linear DDMs, but with time-varying thresholds. If the original neuronal system is nonlinear, one can once again derive a model describing a lower-dimensional diffusion process. The dynamics of the nonlinear DDM can be recast as the motion of a particle on a potential, the general form of which is given analytically for an arbitrary number of alternatives.

## Introduction

Perceptual decision-making tasks require a subject to make a categorical decision based on noisy or ambiguous sensory evidence. A computationally advantageous strategy in doing so is to integrate the sensory evidence in time, thereby improving the signal-to-noise ratio. Indeed, when faced with two possible alternatives, accumulating the difference in evidence for the two alternatives until a fixed threshold is reached is an optimal strategy, in that it minimizes the mean reaction time for a desired level of performance. This is the computation carried out by the sequential probability ratio test devised by Wald [1], and its continuous-time variant, the drift–diffusion model (DDM) [2]. It would be hard to overstate the success of these models in fitting psychophysical data from both animals and human subjects in a wide array of tasks, e.g. [2–5], suggesting that brain circuits can implement a computation analogous to the DDM.

At the same time, neuroscientists have characterized the neuronal activity in cortical areas of monkey, which appear to reflect an integration process during DM tasks [6], although see [7]. The relevant computational building blocks, as revealed from decades of in-vivo electrophysiology, seem to be neurons, the activity of which selectively increases with increasing likelihood for a given upcoming choice. Attractor network models, built on this principle of competing, selective neuronal populations, generate realistic performance and reaction times; they also provide a neuronal description which captures some salient qualitative features of the in-vivo data [8, 9].

While focus in the neuroscience community has been almost exclusively on two-alternative DM (although see [10]), from a computational perspective there does not seem to be any qualitative difference between two or more alternatives. In fact, in a model, increasing the number of alternatives is as trivial as adding another neuronal population to the competition. On the other hand, how to add an alternative to the DDM framework does not seem, on the face of things, obvious. Several groups have sought to link the DDM and attractor networks for two-alternative DM. When the attractor network is assumed linear, one can easily derive an equation for a decision variable, representing the difference in the activities of the two competing populations, which precisely obeys a DDM [11]. For three-alternative DM previous work has shown that a 2D diffusion process can be defined by taking appropriate linear combinations of the three input streams [12]. The general *n*-alternative case for leaky accumulators has also been treated [13]. In the first section of the paper I will summarize and build upon this previous work to illustrate how one can obtain an equivalent DDM, starting from a set of linear firing rate equations which compete through global inhibitory feedback. The relevant decision variables are combinations of the activity of the neuronal populations, and which represent distinct modes of competition. Specifically, I will propose a set of “competition” basis functions which allow for a simple, systematic derivation of the DDMs for any *n*. I will also show how a Bayesian implementation of the the multiple sequential probability ratio test (MSPRT) [14–16] is equivalent in the continuum limit to these same DDMs, but with a moving threshold.

Of course, linear models do not accurately describe the neuronal data from experiments on DM. However, previous work has shown that attractor network models for two-alternative DM operate in the vicinity of pitchfork bifurcation, which is what underlies the winner-take-all competition leading to the decision dynamics [17]. In this regime the neuronal dynamics is well described by a stochastic normal-form equation which right at the bifurcation is precisely equivalent to the DDM with an additional cubic nonlinearity. This *nonlinear* DDM fits behavioral data extremely well, including both correct and error reaction times. In the second part of the paper I will show how such normal-form equations can be derived for an arbitrary number of neuronal populations. These equations can be thought of as nonlinear DDMs and, in fact, are identical to the linear DDMs with the addition of quadratic nonlinearities (for $n>2$). Amazingly, the dynamics of such a nonlinear DDM can be recast as the diffusion of particle on a potential, which is obtained analytically, for arbitrary *n*.

## Results

The canonical drift diffusion model (DDM) can be written

1 $$ \tau \dot{X} = \mu +\xi (t), $$

where *X* is the decision variable, and *μ* is the drift or the degree of evidence in favor of one choice over the other: we can associate choice 1 with positive values of *X* and choice 2 with negative values. The Gaussian process $\xi (t)$ represents noise and/or uncertainty in the integration process, with $\langle \xi (t)\rangle = 0$ and $\langle \xi (t)\xi (t^{\prime })\rangle =\sigma ^{2}\delta (t-t ^{\prime })$. I have also explicitly included a characteristic time scale *τ*, which will appear naturally if one derives Eq. (1) from a neuronal model. The decision variable evolves until reaching one of two boundaries ±*θ* at which point a decision for the corresponding choice has been made.

It is clear that a single variable can easily be used to keep track of two competing processes by virtue of its having two possible signs. But what if there are three or more alternatives? In this case it is less clear. In fact, if we consider a drift–diffusion process such as the one in Eq. (1) as an approximation to an actual integration process carried out by neuronal populations, then there is a systematic approach to deriving the corresponding DDM. The value of such an approach is that one can directly tie the DDM to the neuronal dynamics, thereby linking behavior to neuronal activity.

I will first consider the derivation of a DDM starting from a system of linear firing rate equations. This analysis is similar to that found in Sect. 4.4 of [13], although the model of departure is different. In this case the derivation involves a rotation of the system so as to decouple the linear subspace for the competition between populations from the subspace which describes non-competitive dynamical modes. This rotation is equivalent to expressing the firing rates in terms of a set of orthogonal basis functions: one set for the competition, and another for the non-competitive modes. I subsequently consider a system of nonlinear firing rate equations. In this case one can once again derive a reduced set of equations to describe the decision-making dynamics. The reduced models have precisely the form of the corresponding DDM for a linear system, but now with additional nonlinear terms. These terms reflect the winner-take-all dynamics which emerge in nonlinear systems with multi-stability. Not only do the equations have a simple, closed-form solution for any number of alternatives, but they can be succinctly expressed in terms of a multivariate potential.

### Derivation of a DDM for two-alternative DM

The DDM can be derived from a set of linear equations which model the competition between two populations of neurons, the activity of each of which encodes the accumulated evidence for the corresponding choice. The equations are

2 $$ \begin{aligned} \tau \dot{r}_{1} &= -r_{1}+sr_{1}-cr_{I}+I_{1}+ \xi _{1}(t), \\ \tau \dot{r}_{2} &= -r_{2}+sr_{2}-cr_{I}+I_{2}+ \xi _{2}(t), \\ \tau _{I} \dot{r}_{I} &= -r_{I}+ \frac{g}{2}(r_{1}+r_{2})+I_{I}+\xi _{I}(t), \end{aligned} $$

where $r_{I}$ represents the activity of a population of inhibitory neurons. The parameter *s* represents the strength of excitatory self-coupling and *c* is the strength of the global inhibition. The characteristic time constants of excitation and inhibition are *τ* and $\tau _{I}$ respectively. A choice is made for 1 (2) whenever $r_{1} = r_{th} $ ($r_{2} = r_{th} $).

It is easier to work with the equations if they are written in matrix form, which is
3 where
 In order to derive the DDM, I express the firing rates in terms of three orthogonal basis functions: one which represents a competition between the populations $\mathbf{e}_{1}$, a second for common changes in population rates $\mathbf{e}_{c}$ and a third which captures changes in the activity of the inhibitory cells $\mathbf{e}_{I}$. Specifically I write

4 $$\begin{aligned} \mathbf{r}(t) = \mathbf{e}_{1}X(t)+\mathbf{e}_{C} \mathrm{m}_{C}(t)+ \mathbf{e}_{I}\mathrm{m}_{I}(t), \end{aligned}$$

where $\mathbf{e}_{1} = (1,-1,0)$ and $\mathbf{e}_{C} = (1,1,0)$ and $\mathbf{e}_{I} = (0,0,1)$. The decision variable will be *X*, while $\mathrm{m}_{C}$ and $\mathrm{m}_{I}$ stand for the common mode and inhibitory mode respectively.

The dynamics of each of these modes can be isolated in turn by projecting Eq. (2) onto the appropriate eigenvector. For example, the dynamics for the decision variable *X* are found by projecting onto $\mathbf{e}_{1}$, namely
5 and similarly the dynamics for $\mathrm{m}_{C}$ and $\mathrm{m}_{I}$ and found by projecting onto $\mathbf{e}_{C}$ and $\mathbf{e}_{I}$ respectively. Doing so results in the set of equations

6 $$ \begin{aligned} &\tau \dot{X}= -(1-s)X+\frac{I_{1}-I_{2}}{2}+ \frac{\xi _{1}(t)-\xi _{2}(t)}{2}, \\ &\tau \dot{\mathrm{m}}_{C}= -(1-s)\mathrm{m}_{C}-c \mathrm{m}_{I}+\frac{I_{1}+I_{2}}{2}+\frac{\xi _{1}(t)+\xi _{2}(t)}{2}, \\ &\tau _{I}\dot{\mathrm{m}}_{I}= -\mathrm{m}_{I}+g \mathrm{m}_{C}+I _{I}+\xi _{I}(t). \end{aligned} $$

If the self-coupling sits at the critical value $s = 1$, then these equations simplify to

7 $$ \begin{aligned}& \tau \dot{X}= \frac{I_{1}-I_{2}}{2}+ \frac{\xi _{1}(t)-\xi _{2}(t)}{2}, \\ &\tau \dot{\mathrm{m}}_{C}= -c\mathrm{m}_{I}+ \frac{I_{1}+I_{2}}{2}+\frac{\xi _{1}(t)+\xi _{2}(t)}{2}, \\ &\tau _{I} \dot{\mathrm{m}}_{I}= -\mathrm{m}_{I}+g \mathrm{m}_{c}+I_{I}+\xi _{I}(t), \end{aligned} $$

from which it is clear that the equation for *X* describes a drift–diffusion process. It is formally identical to Eq. (1) with $\mu = \frac{I_{1}-I_{2}}{2}$ and $\xi (t) = \frac{\xi _{1}(t)-\xi _{2}(t)}{2}$. Importantly, *X* is uncoupled from the common and inhibitory modes, which themselves form a coupled subsystem. For $s\ne 1$ the decision variable still decouples from the other two equations, but the process now has a leak term (or ballistic for $s>1$) [18]. It has therefore been argued that obtaining a DDM from linear neuronal models requires fine tuning, a drawback which can be avoided in nonlinear models in which the linear dynamics is approximated via multi-stability; see e.g. [19, 20]. If one ignores the noise terms, the steady state of this linear system is $(X,\mathrm{m}_{C},\mathrm{m}_{I}) = (X_{0},\mathrm{M}_{c},\mathrm{M} _{I})$ where

8 $$ \begin{aligned} \mathrm{M}_{C} &= \frac{I_{1}+I_{2}}{2cg}- \frac{I_{I}}{g}, \\ \mathrm{M}_{I} &= \frac{I_{1}+I_{2}}{2c}. \end{aligned} $$

One can study the stability of this solution by considering a perturbation of the form $(X,\mathrm{m}_{C},\mathrm{m}_{I}) = (X_{0}, \mathrm{M}_{c},\mathrm{M}_{I})+(\delta X,\delta \mathrm{m}_{C},\delta \mathrm{m}_{I})e^{\lambda t}$. Plugging this into Eq. (7) one finds that there is a zero eigenvalue associated with the decision variable, i.e. $\lambda _{1} = 0$, whereas the eigenvalues corresponding to the subsystem comprising the common and inhibitory modes are given by

9 $$ \lambda _{2,3} = -\frac{1}{2\tau _{I}} \biggl(1\pm \sqrt{1-4 \frac{\tau _{I}}{\tau }cg} \biggr), $$

which always have a negative real part. Therefore, as long as $\tau _{I}$ is not too large, perturbations in the common mode or in the inhibition will quickly decay away. This allows one to ignore their dynamics and assume they take on their steady-state values. Finally, the bounds for the decision variable are found by noting that $r_{1} = X+ \mathrm{M}_{C}$ and $r_{2} = -X+\mathrm{M}_{C}$. Therefore, given that the neuronal threshold for a decision is defined as $r_{th} $, we find that $\theta = \pm (r_{th}-\mathrm{M}_{C})$.

### Derivation of a DDM for three-alternative DM

I will go over the derivation of a drift–diffusion process for three-choice DM in some detail for clarity, although conceptually it is very similar to the two-choice case. Then the derivation can be trivially extended to n-alternative DM for any n.

The linear rate equations are once again given by Eq. (3), with


One once again writes the firing rates in terms of orthogonal basis functions, of which there must now be four. The common and inhibitory modes are the same as before, whereas now there will be two distinct modes to describe the competition between the three populations, in contrast to just a single decision variable. Any orthogonal basis in the 2D space for competition is equally valid. However, in order to make the choice systematic, I assume that the first vector is just the one from the two-alternative case, namely $(1,-1,0,0)$, from which it follows that the second must be (up to an amplitude) $(1,1,-2,0)$. Then for *n* alternatives I will always take the first $n-2$ basis vectors to be those from the $n-1$ case. The last eigenvector must be orthogonal to these. Specifically, for $n = 3$, $\mathbf{r} = \mathbf{e}_{1}X _{1}(t)+\mathbf{e}_{2}X_{2}(t)+\mathbf{e}_{C}\mathrm{m}_{C}(t)+ \mathbf{e}_{I}\mathrm{m}_{I}$, where

10 $$ \begin{aligned} &\mathbf{e}_{1}= (1,-1,0,0), \\ &\mathbf{e}_{2}= (1,1,-2,0), \\ &\mathbf{e}_{C}= (1,1,1,0), \\ &\mathbf{e}_{I}= (0,0,0,1). \end{aligned} $$

One projects Eq. (3) onto the four relevant eigenvectors, which leads to the following equations:

11 $$ \begin{aligned} &\tau \dot{X_{1}}= -(1-s)X_{1}+ \frac{I_{1}-I_{2}}{2}+\frac{\xi _{1}(t)- \xi _{2}(t)}{2}, \\ &\tau \dot{X_{2}}= -(1-s)X_{2}+\frac{I_{1}+I_{2}-2I _{3}}{6}+ \frac{\xi _{1}(t)+\xi _{2}(t)-2\xi _{3}(t)}{6}, \\ &\tau \dot{\mathrm{m}_{C}}= -(1-s)\mathrm{m}_{C}-c \mathrm{m}_{I}+\frac{I _{1}+I_{2}+I_{3}}{3}+\frac{\xi _{1}(t)+\xi _{2}(t)+\xi _{3}(t)}{3}, \\ &\tau _{I}\dot{\mathrm{m}_{I}}= -\mathrm{m}_{I}+g \mathrm{m}_{C}+I_{I}+ \xi _{I}(t). \end{aligned} $$

When $s = 1$ then the first two equations in Eq. (11) describe a drift–diffusion process in a two-dimensional subspace, while the coupled dynamics of the common and inhibitory modes are once again strongly damped. The DDM for three-alternative DM can therefore be written

12 $$\begin{aligned} \tau \dot{X}_{1} &= \frac{I_{1}-I_{2}}{2}+ \frac{\xi _{1}(t)-\xi _{2}(t)}{2}, \end{aligned}$$

13 $$\begin{aligned} \tau \dot{X}_{2} &= \frac{I_{1}+I_{2}-2I_{3}}{6}+\frac{\xi _{1}(t)+\xi _{2}(t)-2\xi _{3}(t)}{6}. \end{aligned}$$

Note that the dynamics of the two decision variables $X_{1}$ and $X_{2}$ are coupled through the correlation in their noise sources. The decision boundaries are set by noting that

14 $$ \begin{aligned} &r_{1}= X_{1}+X_{2}+ \mathrm{M}_{C}, \\ &r_{2}= -X_{1}+X_{2}+ \mathrm{M}_{C}, \\ &r_{3}= -2X_{2}+\mathrm{M}_{C}. \end{aligned} $$

Therefore, given that the neuronal threshold for a decision is defined as $r_{th}$ we can set three decision boundaries: 1 Population 1 wins if $X_{2} = -X_{1} +r_{th}-\mathrm{M}_{C}$, 2 Population 2 wins if $X_{2} = X_{1}+r_{th}-\mathrm{M}_{C}$ and 3 Population 3 wins if $X_{2} = -(r_{th}-\mathrm{M}_{C})/2$. These three boundaries define a triangle in (X,Y)-space over which the drift–diffusion process take place.

### Derivation of DDMs for n-alternative DM

The structure of the linear rate equations Eq. (3) can be trivially extended to any number of competing populations. In order to derive the corresponding DDM one need only properly define the basis functions for the firing rates, which was described above. The common and inhibitory modes always have the same structure. If the basis functions are $\mathbf{e}_{i}$ and the corresponding decision variables $X_{i}$ for $i = [1,n-1]$, then the firing rates are $\mathbf{r} = \sum_{i=1}^{n-1}\mathbf{e}_{i}X_{i}(t)$ and it is easy to show that the dynamics for the *k*th decision variable is given by

15 $$ \tau \dot{X}_{k} = \frac{\mathbf{e}_{k}\cdot \mathbf{I}}{\mathbf{e} _{k}\cdot \mathbf{e}_{k}}+ \frac{\mathbf{e}_{k}\cdot \boldsymbol{\xi}}{ \mathbf{e}_{k}\cdot \mathbf{e}_{k}}, $$

as long as $s = 1$. The decision boundaries are defined by setting the firing rates equal to their threshold value for a decision, i.e.

16 $$ \sum_{i=1}^{n-1}\mathbf{e}_{i}X_{i,b} = r_{th}\mathbf{u}, $$

where $\mathbf{u} = (1,1,\ldots,1)$.

The basis set proposed here for *n*-alternative DM is to take for the *k*th eigenvector

17 $$ e_{k} = (1,1,\dots ,1,-k,0,\dots ,0), $$

where the element −*k* appears in the $(k+1)$st spot, and which is a generalization of the eigenvector basis taken earlier for two- and three-alternative DM. With this choice, the equation for the *k*th decision variable can be written

18 $$ \tau \dot{X}_{k} = \frac{\mathbf{e}_{k}\cdot \mathbf{I}}{k+k^{2}}+\frac{ \mathbf{e}_{k}\cdot \boldsymbol{\xi}}{k+k^{2}}. $$

The firing rate for the *i*th *neuronal* population can then be expressed in terms of the decision variables as

19 $$ r_{i} = -(i-1)x_{i-1}+\sum_{l = i}^{n-1}x_{l}+ \mathrm{M}_{C}, $$

which, given a fixed neuronal threshold $r_{th}$, directly gives the bounds on the decision variables. Namely, the *i*th neuronal population wins when $-(i-1)x_{i-1}+\sum_{l = i}^{n-1}x_{l}+\mathrm{M}_{C} > r _{th}$.

**Figure 1:**
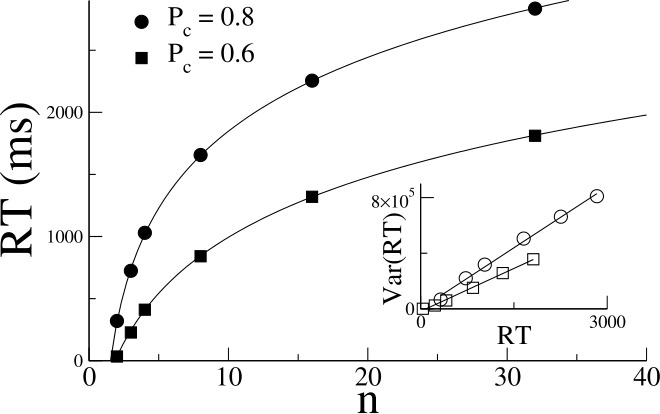
Hick’s Law for multi-alternative DDMs. If the accuracy is held fixed as the number of alternatives *n* is increased, then the mean reaction time increases as the logarithm of *n*. This is shown for both an accuracy of 0.8 (circles) and 0.6 (squares). The inset shows that the variance in the RT increases proportional to the mean RT. Parameter values are $\sigma = 1$, $\tau = 20\text{ ms}$. The thresholds $\theta _{P_{c} = 0.8} = 3$ and $\theta _{P_{c} = 0.6} = 0.9$ for $n = 2$, and are increased for $n>2$ to achieve the same accuracy. The initial condition is always taken to be $X_{i} = 0$ for all *i*. The symbols are the average of 10,000 runs. The solid curves in the main panel are fits to the function $RT = a+b\ln {(c+n)}$. The solid lines in the inset are best-fit linear regressions

The n-alternative DDM reproduces the well-known Hick’s law [21], which postulates that the mean reaction time (RT) increases as the logarithm of the number of alternatives, for fixed accuracy; see Fig. 1.

### DDM for multiple alternatives as the continuous-time limit of the MSPRT

In the previous section I have illustrated how to derive DDMs starting from a set of linear differential equations describing an underlying integration process of several competing data streams. An alternative computational framework would be to apply a statistical test directly to the data, without any pretensions with regards to a neuronal implementation. In fact, the development of an optimal sequential test for multiple alternatives followed closely on the heels of Wald’s work for two alternatives in the 1940s [22]. Subsequent work proposed a Bayesian framework for a multiple sequential probability ratio test (MSPRT) [14–16]. Here I will show how such a Bayesian MSPRT is equivalent to a corresponding DDM in the continuum limit, albeit with moving thresholds.

I will follow the exposition from [16] in setting up the problem, although some notation differs for clarity. I assume that there are *n* possible alternatives, and that the instantaneous evidence for an alternative *i* is given by $z_{i}(t)$, which is drawn from a Gaussian distribution with mean $I_{i}$ and variance $\sigma ^{2}$. The total observed input over all alternatives *n* and up to a time *t* is written *I* and the hypothesis that alternative *i* has the highest mean $H_{i}$. Then, using Bayes theorem, the probability that alternative *i* has the highest mean given the total observed input is

20 $$ \Pr (H_{i}|I) = \frac{\Pr (I|H_{i})\Pr (H_{i})}{\Pr (I)}. $$

Furthermore one has $\Pr (I) = \sum_{k=1}^{n}\Pr (I|H_{k})\Pr (H_{k})$. Given equal priors on the different hypotheses, Eq. (20) can be simplified to

21 $$ \Pr (H_{i}|I) = \frac{\Pr (I|H_{i})}{\sum_{k=1}^{n}\Pr (I|H_{k})}. $$

Finally, the log-likelihood of the alternative *i* having the largest mean up to a time *t* is $L_{i}(t) = \ln {\Pr (H_{i}|I)}$, which is given by

22 $$ L_{i}(t) = \ln {\bigl(\Pr (I|H_{i})\bigr)}-\ln { \Biggl( \sum_{k=1}^{n}e^{\ln {\Pr (I|H _{k})}} \Biggr)}. $$

The choice of Gaussian distributions for the input leads to a simple form for the log-likelihoods. Specifically, the time rate-of-change of the log-likelihood for alternative *i* is given by

23 $$ \dot{L}_{i}(t) = z_{i}-\frac{\sum_{k = 1}^{n}z_{k}e^{y_{k}}}{\sum_{k=1} ^{n}e^{y_{k}}}, $$

where $y_{i}(t) = \int _{0}^{t}dt^{\prime }z_{i}(t^{\prime })$. Note that $z_{i}(t) = I_{i}+\xi _{i}(t)$, where $\xi _{i}$ is a Gaussian white noise process with zero mean and variance $\sigma ^{2}$.

I can now write the $L_{i}$ in terms of the orthogonal basis used in the previous section,

24 $$ \mathbf{L} = \sum_{j = 1}^{n-1} \mathbf{e}_{j}X_{j}(t)+\mathbf{e}_{C}M _{C}(t). $$

Formally projecting onto each mode in turn leads precisely to the DDMs of Eq. (18) (with $\tau = 1$) for the decision variables, while the dynamics for the common mode is

25 $$ \dot{M}_{C} = \frac{1}{n}\sum_{j=1}^{n}z_{j}- \frac{\sum_{k = 1}^{n}z _{k}e^{y_{k}}}{\sum_{k=1}^{n}e^{y_{k}}}. $$

For Bayes-optimal behavior, a choice *i* should be chosen if the log-likelihood exceeds a given threshold, namely if

26 $$ L_{i}(t) = -(i-1)X_{i-1}+\sum_{l = i}^{n-1}X_{l} > \theta - M_{C}(t). $$

Note that the log-likelihood is always negative and hence does not represent a firing rate, as in the differential equations studied in the previous section. This does not pose a problem since we are simply implementing a statistical test. On the other hand, an important difference with the case studied previously is the fact that, for the neuronal models, the common mode was stable and converged to a fixed point. Therefore, the decision variable dynamics was equivalent to the original rate dynamics with a shift of threshold. Here, that is not the case. The common mode represents the normalizing effect of the log-marginal probability of the stimulus, which always changes in time. Specifically, if we assume that $I_{l}>I_{i}$ for all *i*, namely that the mean of the distribution is greatest for alternative *l*, then the expected dynamics of the common mode at long times are

27 $$\begin{aligned} \langle \dot{M}_{C}\rangle =& \frac{1}{n}\sum _{j=1}^{n}I_{j}-\frac{ \sum_{k = 1}^{n}I_{k}e^{I_{k}t}}{\sum_{k=1}^{n}e^{I_{k}t}} \\ \sim & \frac{1}{n}\sum_{j=1}^{n}I_{j}-I_{l} \\ =& -\frac{1}{n}\sum_{j=1}^{n} \vert I_{j}-I_{l} \vert . \end{aligned}$$

**Figure 2:**
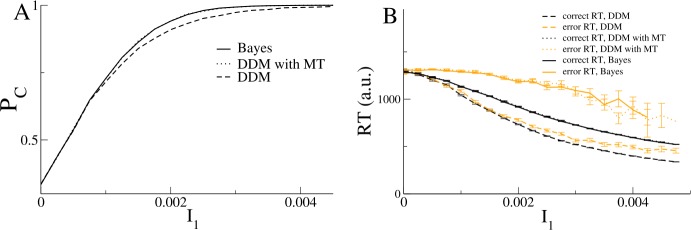
A Bayesian MSPRT is equivalent to DDMs with moving threshold. Accuracy and reaction time for three alternatives. A. Accuracy as a function of the mean of input 1 for the Bayesian test, Eq. (23) (solid line), for the DDM with moving threshold (dotted line) and the DDM with fixed threshold (dashed line). Note that the Bayesian test and the DDM with MT give identical results and are, in fact, mathematically identical. B. Reaction times for the Bayesian test (solid line), DDM with MT (dotted line) and DDM with fixed threshold (dashed line). Orange lines are for error reaction times. Error bars indicate the standard error of the mean. Other parameter values are: $\sigma = 0.03$, $I_{i} = 0$ for $i = 2,3$, $\theta = 1.05$ with MT and $\theta = 1.073$ with fixed threshold. The thresholds were chosen to give identical reaction times for $I_{1} = 0$. The values shown are averages over 10,000 trials for each value of the input

Therefore the DDMs are only equivalent to applying the Bayes theorem if the threshold is allowed to vary in time. In this case they are, in fact, mathematically identical and thus give the same accuracy and reaction time distributions, as shown in Fig. 2.

On the other hand, an equivalent DDM with a fixed threshold has worse accuracy and shorter reaction times. The way I choose the parameters for a fair comparison is to set the fixed threshold such that the mean reaction time is identical to the Bayesian model for zero coherence. Another important difference is that the error RTs are longer than the correct RTs for the Bayesian model, see Fig. 2B, an effect which is commonly seen in experiment (and is also reproduced by the nonlinear DDMs studied in the next section of the paper) [17]. On the other hand correct and error RTs for the DDMs are always the same.

## Derivation of a reduced model for two-choice DM for a nonlinear system

A more realistic firing rate model for a decision-making circuit allows for a nonlinear input-output relationship in neuronal activity. For two-alternative DM the equations are

28 $$ \begin{aligned} &\tau \dot{r}_{1}= -r_{1}+\phi (sr_{1}-cr_{I}+I_{1} )+\xi _{1}(t), \\ &\tau \dot{r}_{2}= -r_{2}+\phi (sr_{2}-cr_{I}+I_{1} )+\xi _{2}(t), \\ &\tau _{I}\dot{r}_{I}= -r_{I}+\phi _{I} \biggl(\frac{g}{2}(r _{1}+r_{2})+I_{I} \biggr)+\xi _{I}(t). \end{aligned} $$

The nonlinear transfer function *ϕ* ($\phi _{I}$) does not need to be specified in the derivation. The noise sources $\xi _{i}$ are taken to be Gaussian white noise and hence must sit outside of the transfer function; they therefore directly model fluctuations in the population firing rate rather than input fluctuations. Input fluctuations can be modeled by allowing for a non-white noise process and including it directly as an additional term in the argument of the transfer function. Note that here I assume the nonlinearity is a smooth function. This is a reasonable assumption for a noisy system such as a neuron or neuronal circuit. Non-smooth systems, such as piecewise linear equations for DM, require a distinct analytical approach; see, e.g., [23].

The details of the derivation for two alternatives can be found in [17], but here I give a flavor for how one proceeds; the process will be similar when there are three or more choices, although the scaling of the perturbative expansion is different. One begins by ignoring the noise sources and linearizing Eq. (28) about a fixed-point value for which the competing populations have the same level of activity, and hence also $I_{1} = I_{2}$. Specifically one takes $(r_{1},r_{2},r_{I}) = (R,R,R_{I})+(\delta r_{1},\delta r_{2}, \delta r_{I})e^{\lambda t}$, where $\delta r\ll 1$. In vector form this can be written $\mathbf{r} = \mathbf{R}+\boldsymbol{\delta}\mathbf{r}e^{\lambda t}$. Plugging this ansatz into Eq. (28) and keeping only terms linear in the perturbations leads to the following system of linear equations:
29 where
30 and the slope of the transfer function $\phi ^{\prime }$ is calculated at the fixed point. Note that the matrix Eq. (30) has a very similar structure to the linear operator in Eq. (3). This system of equations only has a solution if the determinant of the matrix is equal to zero; this yields the characteristic equation for the eigenvalues *λ*. These eigenvalues are

31 $$\begin{aligned} &\lambda _{1}= -\frac{(1-s\phi ^{\prime })}{\tau }, \end{aligned}$$

32 $$\begin{aligned} &\lambda _{2,3}= -\frac{(\tau +\tau _{I}(1-s\phi ^{\prime }))}{2\tau \tau _{I}}\pm \frac{1}{2\tau \tau _{I}}\sqrt {\bigl(\tau -\tau _{I}\bigl(1-s\phi ^{\prime }\bigr) \bigr)^{2}-4 \tau \tau _{I}cg\phi ^{\prime }\phi _{I}^{\prime }}. \end{aligned}$$

Note that $\lambda _{1} = 0$ if $1-s\phi ^{\prime } = 0$, while the real part of the other two eigenvalues is always negative. This indicates that there is an instability of the fixed point in which the activity of the neuronal populations is the same, and that the direction of this instability can be found by setting $1-s\phi ^{\prime } = 0$ and $\lambda = 0$ in Eq. (30). This yields , where
33 the solution of which can clearly be written $\boldsymbol{\delta}\mathbf{r} = (1,-1,0)$. This is the same competition mode as found earlier for the linear system.

### A brief overview of normal-form derivation

At this point it is still unclear how one can leverage this linear analysis to derive a DDM. Specifically, and unlike in the linear case, one cannot simply rotate the system to uncouple the competition dynamics from the non-competitive modes. Also, note that the steady states in a nonlinear system depend on the external inputs, whereas that is not the case in a linear system. In particular, the DDM has a drift term *μ* which ought to be proportional to the difference in inputs $I_{1}-I_{2}$, whereas to perform the linear stability we assumed $I_{1} = I_{2}$. Indeed, if one assumes that the inputs are different, then the fixed-point structure is completely different. The solution is to assume that the inputs are only slightly different, and formalize this by introducing the small parameter *ϵ*. Specifically, we write $I_{1} = I_{0}+\epsilon ^{2}\Delta I +\epsilon ^{3}\bar{I}_{1}$, and $I_{2} = I_{0}+\epsilon ^{2}\Delta I +\epsilon ^{3}\bar{I}_{2}$. In this expansion, $I_{0}$ is the value of the external input which places the system right at the bifurcation in the zero-coherence case. In order to describe the dynamics away from the bifurcation we also allow the external inputs to vary. Specifically, Δ*I* represents the component of the change in input which is common to both populations (overall increased or decreased drive compared to the bifurcation point), while $\bar{I}_{i}$ is a change to the drive to population *i* alone, and hence captures changes in the coherence of the stimulus. The particular scaling of these terms with *ϵ* is enforced by the solvability conditions which appear at each order. That is, the mathematics dictates what these are; if one chose a more general scaling one would find that only these terms would remain.

The firing rates are then also expanded in orders of *ϵ* and written

34 $$\begin{aligned} \mathbf{r} = \mathbf{r_{0}}+\epsilon \mathbf{e_{1}}X(T)+ \mathcal{O}\bigl( \epsilon ^{2}\bigr), \end{aligned}$$

where $\mathbf{r_{0}}$ are the fixed-point values, $\mathbf{e_{1}}$ is the eigenvector corresponding to the zero eigenvalue and *X* is the decision variable which evolves on a slow-time scale, $T = \epsilon ^{2}t$. The slow-time scale arises from the fact that there is an eigenvector with zero eigenvalue; when we change the parameter values slightly, proportional to *ϵ*, the growth rate of the dynamics along that eigenvector is no longer zero, but still very small, in fact proportional to $\epsilon ^{2}$ in this case.

The method for deriving the normal-form equation, i.e. the evolution equation for *X*, involves expanding Eq. (28) in *ϵ*. At each order in *ϵ* there is a set of equations to be solved; at some orders, in this case first at order $\mathcal{O}(\epsilon ^{3})$, the equations cannot be solved and a solvability condition must be satisfied, which leads to the normal-form equation.

### The normal-form equation for two choices

Following the methodology described in the preceding section leads to the evolution equation for the decision variable *X*,

35 $$ \tau \partial _{T}X = \eta (\bar{I}_{1}- \bar{I}_{2})+\mu \Delta IX+ \gamma X^{3}+\xi (t), $$

where for the case of Eq. (28), $\eta = \phi ^{\prime }/2$, $\xi (t) = (\xi _{1}(t)-\xi _{2}(t))/2$ and the coefficients *μ* and *γ* are

36 $$ \begin{aligned} \mu &= \frac{s^{2}\phi ^{\prime \prime }}{cg\phi _{I}^{\prime }}, \\ \gamma &= \frac{s^{3}( \phi ^{\prime \prime })^{2}}{2cg\phi ^{\prime }\phi _{I}^{\prime }}\bigl(s-cg\phi _{I}^{\prime }\bigr)+ \frac{s ^{3}\phi ^{\prime \prime \prime }}{6}, \end{aligned} $$

see [17] for a detailed calculation. Equation (35) provides excellent fits to performance and reaction times for monkeys and human subjects; see Fig. 3 from [17].

It is important to note that the form of Eq. (35) only depends on there being a two-way competition, not on the exact form of the original system. As an example, consider another set of firing rate equations

37 $$ \begin{aligned} \tau \dot{r}_{1} &= -r_{1}+\phi (sr_{1}-cr_{2}+I_{1}), \\ \tau \dot{r} _{2} &= -r_{2}+\phi (sr_{2}-cr_{1}+I_{2}), \end{aligned} $$

where rather than model the inhibition explicitly, an effective inhibitory interaction between the two populations is assumed. In this case the resulting normal-form equation is still Eq. (35). In fact, performing a linear stability analysis on Eq. (37) yields a null eigenvector $e_{1} = (1,-1)$. This indicates that the instability causes one population to grow at the expense of the other, in a symmetric fashion, as before. This is the key point which leads to the normal-form equation. More specifically we see that for both systems $r_{1} = R+X$ while $r_{2} = R-X$, which means that if we flip the sign on the decision variable *X* and switch the labels on the neuronal populations, the dynamics is once again the same. This reflection symmetry ensures that all terms in *X* will have odd powers in Eq. (35) [17]. It is broken only when the inputs to the two populations are different, i.e. by the first term on the r.h.s. in Eq. (35).

As we shall see, the stochastic normal-form equation, Eq. (35), which from now on I will refer to as a nonlinear DDM, has a very different form from the nonlinear DDMs for $n>2$. The reason is, again, the reflection symmetry in the competition subspace for $n=2$, which is not present for $n>2$. Therefore, for $n>2$ the leading-order nonlinearity is quadratic, and, in fact, a much simpler function of the original neuronal parameters.

## Three-alternative forced-choice decision making

The derivation of the normal form, and the corresponding DDM for three-choice DM differs from that for two-alternative DM in several technical details; these differences continue to hold for n-alternative DM for all $n\ge 3$. Therefore I will go through the derivation in some detail here and will then extend it straightforwardly to the other cases.

Again I will make use of a particular system of firing rate equations to illustrate the derivation. I take a simple extension of the firing rate equations for two-alternative DM Eq. (28). The equations are

38 $$ \begin{aligned} &\tau \dot{r}_{1}= -r_{1}+\phi (sr_{1}-cr_{I}+I_{1} )+\xi _{1}(t), \\ &\tau \dot{r}_{2}= -r_{2}+\phi (sr_{2}-cr_{I}+I_{2} )+\xi _{2}(t), \\ &\tau \dot{r}_{3}= -r_{3}+\phi (sr_{3}-cr_{I}+I_{3} )+\xi _{3}(t), \\ &\tau _{I}\dot{r}_{I}= -r_{I}+\phi _{I} \biggl( \frac{g}{3}(r_{1}+r_{2}+r_{3})+I_{I} \biggr)+\xi _{I}(t). \end{aligned} $$

I first ignore the noise terms and consider the linear stability of perturbations of the state in which all three populations have the same level of activity (and so $I_{1} = I_{2} = I_{3} = I_{0}$), i.e. $\mathbf{r} = \mathbf{R}+\boldsymbol{\delta}\mathbf{r}e^{\lambda t}$, where $\mathbf{R} = (R,R,R,R_{I})$. This once again leads to a set of linear equations . The fourth-order characteristic equation leads to precisely the same eigenvalues as in the two-choice case, Eqs. (31) and (32), with the notable difference that the first eigenvalue has multiplicity two. This means that if $1-s\phi ^{\prime } = 0$ then there will be two eigenvalues identically equal to zero and two stable eigenvalues. This is the first indication that the integration process underlying the DM process for three choices will be two-dimensional. The eigenvectors for the DM process are found by solving , where
39 There are many possible solutions; a simple choice would be $\mathbf{e}_{1} = (1,-1,0,0)$ and $\mathbf{e}_{2} = (1,1,-2,0)$, and so $\mathbf{e}_{1}^{T}\cdot \mathbf{e}_{2} = 0$. Note that in this linear subspace any valid choice of eigenvector will have the property that the sum of all the elements will equal zero; this will be true whatever the dimensionality of the DM process and reflects the fact that all of the excitatory populations excite the inhibitory interneurons in equal measure.

To derive the normal form I once again assume that the external inputs to the three populations differ by a small amount, namely $(I_{1},I _{2},I_{3}) = (I_{0},I_{0},I_{0})+\epsilon ^{2}(\bar{I}_{1},\bar{I} _{2},\bar{I}_{3})$, and then expand the firing rates as $\mathbf{r} = \mathbf{R}+\epsilon (\mathbf{e}_{1}X_{1}(T)+\mathbf{e}_{2}X_{2}(T) )+\mathcal{O}(\epsilon ^{2})$, where the slow time is $T = \epsilon t$. Note that the inputs are only expanded to second order in *ϵ*, as opposed to third order as in the previous section. The reason is that the solvability condition leading to the normal-form equation for the 2-alternative case arises at third order. This is due to the fact that the bifurcation has a reflection symmetry, i.e. it is a pitchfork bifurcation and so only odd terms in the decision variable are allowed. The lowest-order nonlinear term is therefore the cubic one. On the other hand, for more than two alternatives there is no such reflection symmetry in the corresponding bifurcation to winner-take-all behavior. Therefore the lowest-order nonlinear term is quadratic, as in a saddle-node bifurcation.

I expand Eq. (38) in orders of *ϵ*. In this case a solvability condition first arises at order $\epsilon ^{2}$, which also accounts for the different scaling of the slow time compared to two-choice DM. Note that there are two solvability conditions, corresponding to eliminating the projection of terms at that order onto both of the left-null eigenvectors of . As before, the left-null eigenvectors are identical to the right-null eigenvectors. Applying the solvability condition yields the normal-form equations

40 $$ \begin{aligned}& \tau \dot{X}_{1}= \frac{\phi ^{\prime }}{2}( \bar{I}_{1}-\bar{I}_{2})+s^{2} \phi ^{\prime \prime }X_{1}X_{2}+\frac{1}{2}\bigl(\xi _{1}(t)-\xi _{2}(t)\bigr), \\ &\tau \dot{X}_{2}= \frac{\phi ^{\prime }}{6}(\bar{I}_{1}+ \bar{I}_{2}-2\bar{I} _{3})+\frac{s^{2}\phi ^{\prime \prime }}{6} \bigl(X_{1}^{2}-3X_{2}^{2}\bigr) + \frac{1}{6}\bigl(\xi _{1}(t)+\xi _{2}(t)-2\xi _{3}(t)\bigr). \end{aligned} $$

**Figure 3:**
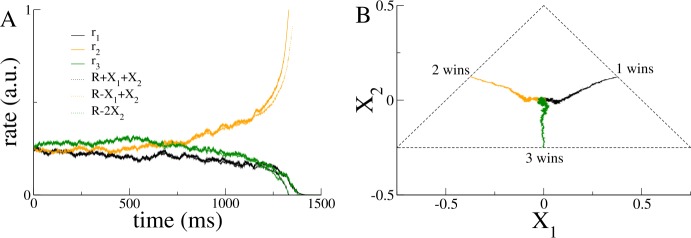
Decision-making dynamics for three alternatives. (**A**) A comparison of the dynamics from integration of the coupled firing rate equations Eq. (38) (solid lines) and from the DDM, Eq. (40) (dotted lines). (**B**) The dynamics from three separate simulations of the DDM, Eq. (40), indicating three different winning alternatives. Parameters are $\tau = 20\text{ ms}$, $\tau _{I} = 10\text{ ms}$, $s = c = g = $1, $\sigma = 0.01$. The function $\phi (x) = 0$ for $x<0$, $x^{2}$ for $0\le x\le 1$ and $2 \sqrt{x-3/4}$ for $x>1$. For this choice of transfer function and these parameter values, there is a bifurcation for $I_{0} = 1/2$, which is the value taken here. The steady-state firing rate $R = 1/4$. The threshold $\theta = 1/2$

The nonlinear DDM Eq. (40) provides an asymptotically correct description of the full dynamics in Eq. (38) in the vicinity of the bifurcation leading to the decision-making behavior. Figure 3(A) shows a comparison of the firing rate dynamics with the nonlinear DDM right at the bifurcation. The appropriate combinations of the two decision variables $X_{1}$ and $X_{2}$ clearly track the rate dynamics accurately, including the correct choice (here population 2) and reaction time. The nonlinear drift–diffusion process evolves in a triangular section of the plane; see Fig. 3(B).

### A note on the difference between the nonlinear DDM for 2A and 3A DM

The dynamics of the nonlinear DDM for 2A, Eq. (35), depends strongly on the sign of the cubic coefficient *γ*. Specifically, when $\gamma < 0$ the bifurcation is supercritical, while for $\gamma > 0$ it is subcritical, indicating the existence of a region of multi-stability for $\Delta I < 0$. In fact, in experiment, cells in parietal cortex which exhibit ramping activity during perceptual DM tasks, also readily show delay activity in anticipation of the sacade to their response field [24]. One possible mechanism for this would be precisely this type of multi-stability. When $\Delta I = 0$, i.e. when the system sits squarely at the bifurcation, Eq. (35) is identical to its linear counterpart with the sole exception of the cubic term. For $\gamma < 0$ the state $X = 0$ is stabilized. In fact, the dynamics of the decision variable can be viewed as the motion of a particle in a potential, which for $\gamma < 0$ increases rapidly as X grows, pushing the particle back. On the other hand, for $\gamma > 0$ the potential accelerates the motion of *X*, pushing it off to ±∞. This is very similar to the potential for the linear DDM with absorbing boundaries. Therefore, the nonlinear DDM for two-alternatives is qualitatively similar to the linear DDM when it is subcritical, and hence when the original neuronal system is multi-stable.

On the other hand, the nonlinear DDM for three alternatives, Eq. (40), has a much simpler, quadratic nonlinearity. The consequence of this is that there are no stable fixed points and the decision variables always evolve to ±∞. Furthermore, to leading order there is no dependence on the mean input, indicating that the dynamics is dominated by the behavior right at the bifurcation.^1^ The upshot is that Eq. (40) is as similar to the corresponding linear DDM with absorbing boundaries as possible for a nonlinear system without fine tuning. This remains true for all $n>2$.

This also means that neuronal systems with inhibition-mediated winner-take-all dynamics are generically multi-stable for $n>2$, although for $n = 2$ they need not be. This is due to the reflection symmetry present only for $n = 2$.

## *n*-alternative forced-choice decision making

One can now extend the analysis for three-alternative DM to the more general *n*-choice case. Again I start with a set of firing rate equations

$$ \begin{aligned} &\tau \dot{r}_{1}= -r_{1}+\phi (sr_{1}-cr_{I}+I_{1} )+\xi _{1}(t), \\ &\tau \dot{r}_{2}= -r_{2}+\phi (sr_{2}-cr_{I}+I_{2} )+\xi _{2}(t), \\ &\vdots \\ &\tau \dot{r}_{n}= -r_{n}+\phi (sr_{n}-cr _{I}+I_{n} )+\xi _{n}(t), \\ &\tau _{I}\dot{r}_{I}= -r_{I}+\phi _{I} \Biggl(\frac{g}{n}\sum_{j=1}^{n}r_{j}+I_{I} \Biggr)+\xi _{I}(t). \end{aligned} $$

A linear stability analysis shows that the eigenvalues of perturbations of the state $\mathbf{r} = (R,R,\ldots ,R,R_{I})$ are given by Eqs. (31) and (32), where the first eigenvalue has multiplicity $n-1$. Therefore the decision-making dynamics evolves on a manifold of dimension $n-1$. The linear subspace associated with this manifold is spanned by $n-1$ eigenvectors which are mutually orthogonal and the elements of which sum to zero. For *n* alternatives, we take $n-1$ eigenvectors of the form $e_{k} = (1,1,\dots ,1,-k,0, \dots ,0)$, for the *k*th eigenvector (again, the −*k* sits in the (k+1)-st spot). Therefore one can write

41 $$ \mathbf{r} = \mathbf{R}+\epsilon \sum_{i=1}^{n-1} \mathbf{e} _{i}X_{i}(T)+\mathcal{O}\bigl(\epsilon ^{2}\bigr). $$

Following the same procedure as in the case of three-choice DM and applying the $n-1$ solvability conditions at order $\mathcal{O}( \epsilon ^{2})$, one arrives at the following normal-form equation for the *k*th decision variable for *n* alternatives:

42 $$ \begin{aligned}[b] \tau \dot{X}_{k} ={} & \phi ^{\prime } \frac{\mathbf{e}_{k}^{T}\cdot \mathbf{I}}{k+k ^{2}}+\frac{s^{2}\phi ^{\prime \prime }}{2(k+k^{2})} \Biggl(\sum_{j=1}^{k-1} \bigl(j+j^{2}\bigr)X _{j}^{2}-k \bigl(k^{2}-1\bigr)X_{k}^{2}+2\bigl(k+k^{2} \bigr)X_{k}\sum_{j=k+1}^{n-1}X_{j} \Biggr) \\ &{}+\frac{\mathbf{e}_{k}^{T}\cdot \boldsymbol{\xi}(\mathbf{t})}{k+k^{2}}. \end{aligned} $$

It is illuminating to write this formula out explicitly for some of the decision variables:

43 $$ \begin{aligned} &\tau \dot{X}_{1}= \phi ^{\prime } \frac{1}{2}(\bar{I}_{1}-\bar{I}_{2})+s ^{2} \phi ^{\prime \prime }X_{1}\sum_{j=2}^{n-1}X_{j}, \\ &\tau \dot{X}_{2}= \phi ^{\prime }\frac{1}{6}( \bar{I}_{1}+\bar{I}_{2}-2\bar{I}_{3})+ \frac{s^{2}\phi ^{\prime \prime }}{6} \Biggl(X_{1}^{2}-3X_{2}^{2}+6X_{2} \sum_{j=3}^{n-1}X_{j} \Biggr), \\ &\tau \dot{X}_{3}= \phi ^{\prime }\frac{1}{12}( \bar{I}_{1}+\bar{I}_{2}+ \bar{I}_{3}-3 \bar{I}_{4})+\frac{s^{2}\phi ^{\prime \prime }}{12} \Biggl(X_{1}^{2}+3X _{2}^{2}-12X_{3}^{2}+12X_{3} \sum_{j=4}^{n-1}X_{j} \Biggr), \\ & \vdots \\ &\tau \dot{X}_{n-1}= \phi ^{\prime }\frac{1}{n(n-1)}\Biggl(\sum _{j=1}^{n-1} \bar{I}_{j}-(n-1) \bar{I}_{n}\Biggr)\\ &\hphantom{\tau \dot{X}_{n-1}=}{}+\frac{s^{2}\phi ^{\prime \prime }}{n(n-1)} \Biggl( \sum _{j=1}^{n-2}\bigl(j+j^{2} \bigr)X_{j}^{2}-n(n-1)^{2}X_{n}^{2} \Biggr), \end{aligned} $$

where I have left off the noise terms for simplicity.

Surprisingly, the $n-1$ equations for the decision variables in *n*-alternative DM can all be derived from a single, multivariate function:

44 $$ f(X_{1},\dots ,X_{n}) = -a\sum _{j=1}^{n-1}\langle \mathbf{e}_{j} \cdot \mathbf{I}\rangle X_{j}-\frac{b}{2} \Biggl(\sum _{j=1}^{n-2}\bigl(j+j ^{2} \bigr)X_{j}^{2}\sum_{i=j+1}^{n-1}X_{i}- \sum_{j=1}^{n-1} \frac{j(j^{2}-1)}{3}X_{j}^{3} \Biggr). $$

For the system of firing rate equations studied here the parameters $a = \phi ^{\prime }$ and $b = s^{2}\phi ^{\prime \prime }$. Then the equation for the *k*th decision variable is simply

45 $$ \tau \dot{X}_{k} = -\frac{1}{k+k^{2}} \frac{\partial f}{\partial X_{k}} + \frac{\mathbf{e}_{k}\cdot \xi (t)}{k+k ^{2}} . $$

The dynamics of the function *f* is given by

46 $$ \begin{aligned}[b] \tau \frac{\partial f}{\partial t} &= \sum _{j=1}^{n-1}\frac{\partial f}{\partial X_{j}}\dot{X}_{j} \\ &= -\sum_{j=1}^{n-1}\frac{1}{k+k^{2}} \biggl(\frac{\partial f}{\partial X_{j}} \biggr)^{2} < 0, \end{aligned} $$

where I have ignored the effect of noise. Therefore the dynamics of the decision variables can be thought of as the motion of a particle on a potential landscape, given by *f*. Noise sources lead to a diffusion along this landscape.

## Discussion

In this paper I have illustrated how to derive drift–diffusion models starting from models of neuronal competition for n-alternative decision-making tasks. In the case of linear systems, the derivation consists of nothing more than a rotation of the dynamics onto a subspace of competition modes. This idea is not new, e.g. [13], although I have made the derivation explicit here, and have chosen as a model of departure one in which inhibition is explicitly included as a dynamical variable. It turns out that a Bayesian implementation of a multiple sequential probability ratio test is also equivalent to a DDM in the continuum limit, albeit with time-varying thresholds.

For nonlinear systems, the corresponding DDM is a stochastic normal form, which is obtained here using the method of multiple-scales [25]. The nonlinear DDM was obtained earlier for the special case of two-alternative DM [17]. For four-alternative DM the nonlinear DDM was obtained with a different set of competitive basis functions [26] to describe performance and reaction time from experiments with human subjects. This led to a different set of coupled normal-form equations from those given by Eq. (42), although the resulting dynamics is, of course, the same. The advantage of the choice I have made in this paper for the basis functions, is that they are easily generalizable for any *n*, leading to a simple, closed-form expression for the nonlinear DDM for any arbitrary number of alternatives, Eq. (42).

An alternative approach to describing the behavior in DM tasks, is to develop a statistical or probabilistic description of evidence accumulation; see, e.g., [1, 13, 15, 22, 27]. Such an approach also often leads to a drift–diffusion process in some limit, as is the case for the Bayesian MSPRT studied here, and see also [28]. In fact, recent work has shown that an optimal policy for multiple-alternative decision making can be approximately implemented by an accumulation process with time-varying thresholds, similar to the Bayesian model studied in this manuscript [29]. From a neuroscience perspective, however, it is of interest to pin down how the dynamics of neuronal circuits might give rise to animal behavior which is well described by a drift–diffusion process. This necessitates the analysis of neuronal models at some level. What I have shown here is that the dynamics in a network of *n* neuronal populations which compete via a global pool of inhibitory interneurons, can in general be formally reduced to a nonlinear DDM of dimension $n-1$. The nonlinear DDMs differ from the linear DDMs through the presence of quadratic (or cubic for $n = 2$) nonlinearities which accelerate the winner-take-all competition. In practical terms this nonlinear acceleration serves the same role as the hard threshold in the linear DDMs. Therefore the two classes of DDMs have quite similar behavior.

The DDM is one of the most-used models for fitting data from two-alternative forced-choice decision-making experiments. In fact it provides fits to decision accuracy and reaction time in a wide array of tasks, e.g. [2, 3, 30]. Here I have illustrated how the DDM can be extended to *n* alternatives straightforwardly. It remains to be seen if such DDMs will fit accuracy and reaction times as well as their two-alternative cousin, although one may refer to promising results from [12] for three alternatives. Note also that the form of the nonlinear DDMs, Eqs. (44) and (45) does not depend on the details of the original neuronal equations; this is what is meant by a normal-form equation. The only assumptions needed for the validity of the normal-form equations are that there be global, nonlinear competition between *n* populations. Of course, if the normal form is derived from a given neuronal model, then the parameters *a* and *b* of the nonlinear potential Eq. (44) will depend on the original neuronal parameters.

As stated earlier, the nonlinear DDMs can have dynamics quite similar to the standard, linear DDM with hard thresholds. Nonetheless, there are important qualitative differences between the two classes of models. First of all, both correct and error reaction-time distributions are identical in the linear DDMs, given unbiased initial conditions, whereas the nonlinear DDMs generically show longer error reaction times [17], also a feature of the Bayesian MSPRT. Because error reaction times in experiment indeed tend to be longer than correct ones, the linear DDM cannot be directly fit to data. Rather, variability in the drift rate across trials can be assumed in order to account for differences in error and correct reaction times; see, e.g., [30]. Secondly, nonlinear DDMs exhibit intrinsic dynamics which reflect the winner-take-all nature of neuronal models with strong recurrent connectivity. As a consequence, as the decision variables increase (or decrease) from their initial state, they undergo an acceleration which does not explicitly depend on the value of the external input. This means that the response of the system to fluctuations in the input is not the same late in a trial as it is early on. Specifically, later fluctuations will have lesser impact. Precisely this effect has been seen in the response of neurons in parietal area LIP in monkeys in two-alternative forced-choice decision-making experiments; see Fig. 10B in [31]. Given that network models of neuronal activity driving decision-making behavior lead to nonlinear DDMs, fitting such models to experimental data in principle allows one to link behavioral measures to the underlying neuronal parameters. In fact, it may be that the linear DDM has been so successful in fitting behavioral data over the years precisely because it is a close approximation to the true nonlinear DDM which arises in neuronal circuits with winner-take-all dynamics.

## Appendix

### 1.1 Derivation of the normal form for three-alternative DM

I assume the inputs to the three competing neuronal populations in Eq. (38) are slightly different, namely $I_{i} = I _{0}+\epsilon ^{2}\bar{I}_{i}$ for $i = \{1,2,3\}$, which defines the small parameter $\epsilon \ll 1$. The firing rates are expanded as $\mathbf{r} = \mathbf{R}+\epsilon \mathbf{r}_{1}+\epsilon ^{2} \mathbf{r}_{2}+\mathcal{O}(\epsilon ^{3})$ where **R** are the values of the rates at the fixed point. Finally, we define a slow time $T = \epsilon t$. Plugging the expansions for the inputs and rates into Eq. (38) and expanding leads to the following system of equations:

47

where to place the system at the bifurcation to winner-take-all behavior I choose $1-s\phi ^{\prime } = 0$. Then we have the linear operator

48

and

49 $$\begin{aligned}& \mathbf{L}_{1} = \begin{pmatrix} \tau \partial _{T} & 0 & 0 & 0 \\ 0 & \tau \partial _{T} & 0 & 0 \\ 0 & 0 & \tau \partial _{T} & 0 \\ 0 & 0 & 0 & \tau _{I}\partial _{T} \end{pmatrix}, \end{aligned}$$

50 $$\begin{aligned}& \mathbf{N}_{2} = \phi ^{\prime } \begin{pmatrix} \bar{I}_{1} \\ \bar{I}_{2} \\ \bar{I}_{3} \\ 0 \end{pmatrix}+ \frac{1}{2} \begin{pmatrix} \phi ^{\prime \prime }(sr_{11}-cr_{I1})^{2} \\ \phi ^{\prime \prime }(sr_{21}-cr_{I1})^{2} \\ \phi ^{\prime \prime }(sr_{31}-cr_{I1})^{2} \\ \phi _{I}^{\prime \prime }\frac{g^{2}}{9}(r_{11}+r_{21}+r_{31})^{2} \end{pmatrix}. \end{aligned}$$

Now one solves the equations order by order. At $\boldsymbol{\mathcal{O}(\epsilon )}$

51

The solution can be written $\mathbf{r}_{1} = \mathbf{e}_{1}X_{1}(T)+ \mathbf{e}_{2}X_{2}(T)$, where the right-null eigenvectors of  are $\mathbf{e}_{1} = (1,-1,0,0)$ and $\mathbf{e}_{2} = (1,1,-2,0)$. The left-null eigenvectors, which satisfy  are identical to the right-null eigenvectors in this case. Up to $\boldsymbol{\mathcal{O}(\epsilon ^{2})}$:

52

Given the solution $r_{1}$, the forcing term has the form

53 $$ \mathbf{N}_{2} = \phi ^{\prime } \begin{pmatrix} \bar{I}_{1} \\ \bar{I}_{2} \\ \bar{I}_{3} \\ 0 \end{pmatrix} + \frac{s^{2}\phi ^{\prime \prime }}{2} \begin{pmatrix} (X_{1}+X_{2})^{2} \\ (-X_{1}+X_{2})^{2} \\ 4X_{2}^{2} \\ 0 \end{pmatrix} . $$

This forcing term contains a projection in the left-null eigenspace of . This projection cannot give rise to a solution in $\mathbf{r}_{2}$ and so must be eliminated. The solvability conditions are therefore

54

which yield Eq. (40). Note that the resulting normal form has detuning or bias terms, proportional to differences in inputs; these would be called ‘drifts’ in the context of a DDM, but no term linearly proportional to the normal-form amplitudes, or ‘decision variables’, as in the normal form for two-choice DM. Such a term describes the growth rate of the critical mode away from the bifurcation point: attracting to one side and repelling to the other. To derive these terms we will need to continue the calculation to next order. First, we must solve for $\mathbf{r}_{2}$. We note that, while $\mathbf{r}_{2}$ cannot have components in the directions $\mathbf{e}_{1}$ or $\mathbf{e}_{2}$, the components in the directions $\mathbf{e}_{C} = (1,1,1,0)$ and $\mathbf{e}_{I} = (0,0,0,1)$ (the common and inhibitory modes which we discussed in the section on DDMs) can be solved for.

Therefore we take $\mathbf{r}_{2} = \mathbf{e}_{C}R_{2C}+\mathbf{e} _{I}R_{2I}$ and project

55

which yields

56 $$ R_{2C} = \frac{1}{g\phi _{I}^{\prime }}R_{2I},\qquad R_{2I} = \frac{1}{3c}(\bar{I} _{1}+\bar{I}_{2}+ \bar{I}_{3})+\frac{s^{2}\phi ^{\prime \prime }}{3c\phi ^{\prime }}\bigl(X_{1} ^{2}-X_{2}^{2}\bigr). $$

Up to $\boldsymbol{\mathcal{O}(\epsilon ^{3})}$:

57

where

58 $$\begin{aligned} \mathbf{N}_{3} =& \begin{pmatrix} s\phi ^{\prime \prime }(X_{1}+X_{2}) ((s-cg\phi _{I}^{\prime })R_{2C}+\bar{I}_{1} ) \\ s\phi ^{\prime \prime }(-X_{1}+X_{2}) ((s-cg\phi _{I}^{\prime })R_{2C}+\bar{I}_{2} ) \\ -2s\phi ^{\prime \prime }X_{2} ((s-cg\phi _{I}^{\prime })R_{2C}+\bar{I}_{3} ) \\ 0 \end{pmatrix} \\ &{}+ \frac{s^{3}\phi ^{\prime \prime \prime }}{6} \begin{pmatrix} (X_{1}+X_{2})^{3} \\ (-X_{1}+X_{2})^{3} \\ -8X_{2}^{3} \\ 0 \end{pmatrix} . \end{aligned}$$

Applying the solvability conditions gives the equations

59 $$ 0 = \mathbf{e}_{1}\cdot \mathbf{N}_{3},\qquad 0 = \mathbf{e}_{2}\cdot \mathbf{N}_{3}. $$

If we add the resulting terms to the normal-form equations derived at $\mathcal{O}(\epsilon ^{2})$ we have

60 $$ \begin{aligned} \tau \partial _{T}X_{1} ={}& \frac{\phi ^{\prime }}{2}(\bar{I}_{1}-\bar{I}_{2})+s \phi ^{\prime \prime } \biggl(\frac{\bar{I}_{1}+\bar{I}_{2}}{2}+\frac{(s-cg\phi _{I} ^{\prime })}{3cg\phi _{I}^{\prime }}( \bar{I}_{1}+\bar{I}_{2}+\bar{I}_{3}) \biggr)X _{1} \\ &{}+\frac{s\phi ^{\prime \prime }}{2}(\bar{I}_{1}-\bar{I}_{2})X_{2}+s \phi ^{\prime \prime }X _{1}X_{2} \\ &{}+\frac{(s-cg\phi _{I}^{\prime })}{3cg\phi ^{\prime }\phi _{I}^{\prime }}s^{3}\bigl( \phi ^{\prime \prime } \bigr)^{2}X_{1}\bigl(X_{1}^{2}-X_{2}^{2} \bigr)+\frac{s^{3}\phi ^{\prime \prime \prime }}{6}X _{1}\bigl(X_{1}^{2}+3X_{2}^{2} \bigr), \\ \tau \partial _{T}X_{2} ={}& \frac{\phi ^{\prime }}{6}( \bar{I}_{1}+\bar{I}_{2}-2\bar{I}_{3})+s\phi ^{\prime \prime } \biggl(\frac{ \bar{I}_{1}+\bar{I}_{2}+4\bar{I}_{3}}{6}+\frac{(s-cg\phi _{I}^{\prime })}{3cg \phi _{I}^{\prime }}( \bar{I}_{1}+\bar{I}_{2}+\bar{I}_{3}) \biggr)X_{2}\\ &{} +\frac{s \phi ^{\prime \prime }}{6}(\bar{I}_{1}+ \bar{I}_{2})X_{1}+ \frac{s^{2}\phi ^{\prime \prime }}{6}\bigl(X_{1}^{2}-3X_{2}^{2} \bigr) \\ &{}+\frac{(s-cg\phi _{I} ^{\prime })}{3cg\phi ^{\prime }\phi _{I}^{\prime }}s^{3}\bigl(\phi ^{\prime \prime } \bigr)^{2}X_{2}\bigl(X_{1}^{2}-X _{2}^{2}\bigr)+\frac{s^{3}\phi ^{\prime \prime \prime }}{6}X_{2} \bigl(X_{1}^{2}+3X_{2}^{2}\bigr). \end{aligned} $$

#### 1.1.1 Normal form for three-choice DM starting with a different neuronal model

If we consider a different neuronal model for three-choice DM we can still arrive at the same normal-form equations. The only important factor is the presence of a linear subspace associated with a zero eigenvalue of multiplicity two and which is spanned by eigenvectors representing competition modes. As an example, consider the model

61 $$ \begin{aligned} \tau \dot{r}_{1} &= -r_{1}+\phi \biggl(sr_{1}-\frac{c}{2}(r_{2}+r_{3})+I _{1} \biggr), \\ \tau \dot{r}_{2} &= -r_{2}+\phi \biggl(sr_{2}- \frac{c}{2}(r _{1}+r_{3})+I_{2} \biggr), \\ \tau \dot{r}_{3} &= -r_{3}+\phi \biggl(sr_{3}- \frac{c}{2}(r_{1}+r_{2})+I_{3} \biggr), \end{aligned} $$

in which the inhibition is not explicitly modeled as before. A linear stability analysis reveals that there is a zero eigenvalue with multiplicity two when $\frac{c\phi ^{\prime }}{2} = 1-s\phi ^{\prime }$ and $1-s\phi ^{\prime }> 0$. Therefore, the instability mechanism here is the cross-inhibition, while before it was the self-coupling. Nonetheless, it is only the structure of the corresponding eigenvectors which matters. In this case the two eigenvectors with zero eigenvalue are $\mathbf{e}_{1} = (1,-1,0)$ and $\mathbf{e}_{2} = (1,1,-2)$. Following the methodology described above leads to the identical normal-form equations Eq. (40) with *s* replaced by $s+c/2$.

### 1.2 Normal-form equations for n-choice DM

A neuronal model for n-choice DM $(n>3)$ is

62 $$ \begin{aligned}& \tau \dot{r}_{1}= -r_{1}+\phi (sr_{1}-cr_{I}+I_{1} )+\xi _{1}(t), \\ &\tau \dot{r}_{2}= -r_{2}+\phi (sr_{2}-cr_{I}+I_{2} )+\xi _{2}(t), \\ &\vdots \\ &\tau \dot{r}_{n}= -r_{n}+\phi (sr_{n}-cr _{I}+I_{n} )+\xi _{n}(t), \\ &\tau _{I}\dot{r}_{I}= -r_{I}+\phi _{I} \Biggl(\frac{g}{n}\sum_{j=1}^{n}r_{j}+I_{I} \Biggr)+\xi _{I}(t). \end{aligned} $$

I again consider small differences in the inputs, $I_{i} = I_{0}+ \epsilon ^{2}\Delta I_{i}$ for $i = [1,n]$, expand the rates as $\mathbf{r} = \mathbf{R}+\epsilon \mathbf{r}_{1}+\mathcal{O}(\epsilon ^{2})$, where **R** are the rates at the fixed point, and define the slow time $T = \epsilon t$. If we take $1-s\phi ^{\prime } = 0$ then the matrix of the linearized system has a zero eigenvalue with multiplicity $n-1$. Carrying out an expansion up to $\mathcal{O}(\epsilon ^{2})$ as described above and applying the $n-1$ solvability conditions leads to the normal-form equations Eq. (42).

## Footnotes

1 Changing the mean input leads to higher-order corrections which can be calculated straightforwardly; see the Appendix.

## Abbreviations

DDM: Drift Diffusion Model
DM: Decision Making
